# Bilateral synchronous adrenal metastases of renal cell carcinoma: A case report and review of the literature

**DOI:** 10.3892/ol.2015.2915

**Published:** 2015-01-28

**Authors:** HAKAN ÖZTÜRK

**Affiliations:** Department of Urology, School of Medicine, Sifa University, Izmir 35240, Turkey

**Keywords:** renal cell carcinoma, adrenal metastasis, synchronous metastasis, diagnosis, treatment

## Abstract

Renal cell carcinomas (RCCs) metastasize to the adrenal glands via various mechanisms, including lymphatic vessel arterial embolism and retrograde venous embolism. The rate of ipsilateral metastasis is 3–5% and the rate of contralateral metastasis is ~0.7%, however, synchronous bilateral adrenal metastases are extremely rare. Therefore, the optimal diagnosis and treatment strategy for this condition is yet to be thoroughly defined. In the present study, a 50-year-old male patient presented with right flank pain. Ultrasonography (US) revealed a right renal mass and bilateral adrenal metastases, and a computerized tomography (CT) scan determined the size of the lesions: An 86×83×66-mm mass in the lower pole of the right kidney, an 18×12×10-mm mass in the right adrenal gland, and a 69×51×53-mm mass in the left adrenal gland with central necrosis and peripheral contrast uptake. A US-guided biopsy was performed which determined a diagnosis of right RCC and bilateral synchronous adrenal metastasis. Immunohistochemical examination of the biopsy revealed clear cell carcinoma (Fuhrman grade, III). Consequently, right radical nephrectomy, right partial adrenalectomy (with frozen section examination) and left adrenalectomy were planned. The bilateral synchronous adrenal metastases posed a challenge in the diagnosis and treatment of the disease, as there is no standard approach in the literature for the treatment of such patients. However, metastasectomy was selected, as it appears to be the most effective treatment strategy for increasing the rate of cancer-specific survival. As an adrenal mass was present in the current patient, a hormonal examination was recommended and an adrenal-preserving minimally invasive surgical procedure using frozen section examination during surgery was particularly important to prevent the patient from developing adrenal insufficiency.

## Introduction

Renal cell carcinomas (RCCs) account for 2–3% of all types of cancer worldwide and the highest incidence rate is observed in developed countries. The disease is more common in males, with a male to female ratio of 1.5:1, and the highest incidence is observed between the ages of 60 and 70 years (1). Furthermore, the five-year survival rate of RCC is 68.4% and the worldwide incidence rate increases by 2% annually (2). RCCs can metastasize to almost every organ, including the lungs (50–60%), liver (30–40%), bones (30–40%) and brain (5%), and ~25% of RCC patients will already have multiple distant metastases at the time of presentation, such as lung, lymph node, liver or bone metastases (3). The rate of ipsilateral adrenal metastasis from RCC ranges from 1.1 to 10% in RCC patients and increases to 6–29% in autopsy series (4,5). By contrast, contralateral adrenal metastases rarely occur (metastasis rate, ~0.7%) and bilateral adrenal metastases are limited to ~20 cases reported in the literature (4). Bilateral adrenal metastases may occur as synchronous or metachronous lesions; however, the management of synchronous adrenal metastases is considerably more challenging. In the majority of RCC metastasis cases, removal of the metastatic mass contributes to the rate of survival; however, bilateral adrenalectomy may expose the patient to novel endocrinological complications, such as the development of iatrogenic Addison’s disease. Therefore, minimally invasive surgical procedures and partial adrenalectomy, in addition to radical nephrectomy, should be the preferred treatment strategy for bilateral synchronous adrenal metastasis from RCC. In the current case, preservation of the right adrenal gland was considered to be more feasible due to the size of the metastatic lesion. Therefore, right partial adrenalectomy was preferred to left partial adrenalectomy during the planning of the surgical procedure. Written informed consent was obtained from the patient, and all procedures were followed in accordance with the ethical standards of the Committee on Human Experimentation, World Medical Association (Izmir, Turkey) and the Declaration of Helsinki 1975, as revised in 2000.

## Case report

### Clinical and radiological features

**Figure 1:**
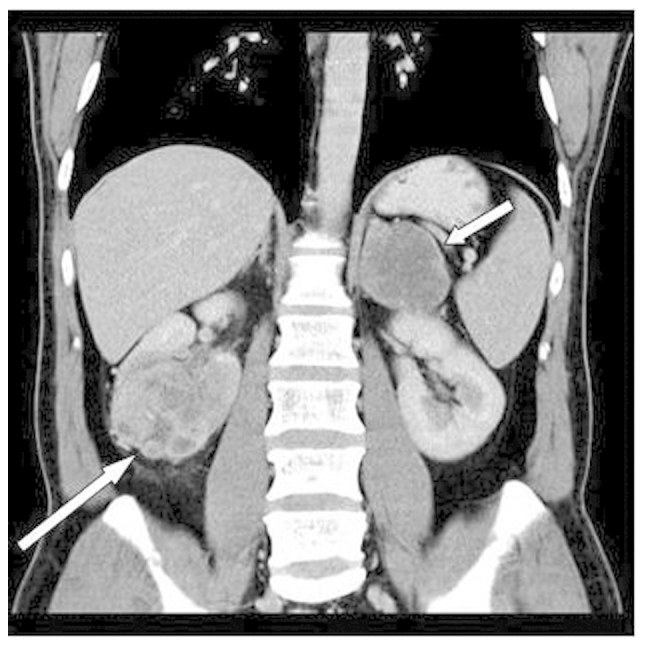
Computed tomography imaging of the coronal reconstruction indicating the right renal mass (long arrow) and the left adrenal metastatic mass (short arrow).

**Figure 2:**
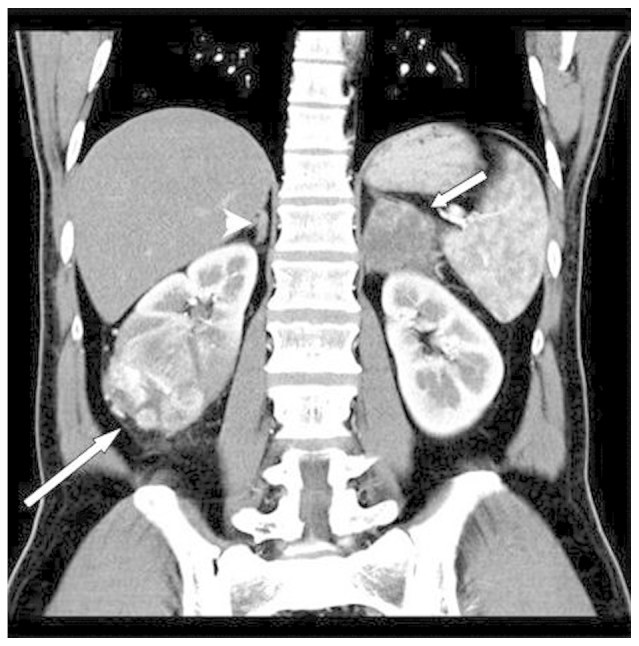
Computed tomography imaging of the coronal reconstruction indicating the right renal mass (long arrow), the right adrenal metastatic mass (arrowhead) and the left adrenal metastatic mass (short arrow).

**Figure 3:**
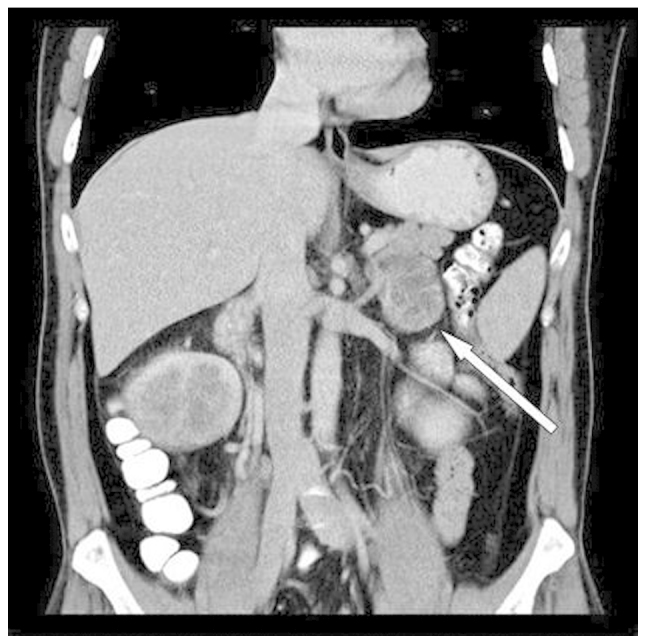
Computed tomography imaging of the coronal reconstruction indicating the left adrenal metastatic mass (arrow).

In April 2014, a 50-year-old male patient was admitted to Basmane Hospital of Sifa University (Basmane, Turkey), presenting with right flank pain. Ultrasonography (US) revealed an 80×81×57-mm mass (Figs. 1 and 2) located in the lower pole of the right kidney with solid-cystic components and heterogeneous echogenicity. Computerized tomography (CT) scans identified a primary mass in the lower pole of the right kidney measuring 86×83×66 mm and the left adrenal metastatic mass (Fig. 1), which extended to the posterior pararenal area in the inferior region, and to the renal pelvis and hilus in the superior region. Furthermore, the metastatic right renal mass demonstrated marked central contrast uptake in the arterial phase. Additionally, two metastatic masses, measuring 69×51×53 mm and 18×12×10 mm, were detected in the left (Fig. 3) and right adrenal gland (Fig. 2), respectively, with central necrosis and peripheral contrast uptake.

Serum adrenocorticotropic hormone (ACTH; 12 pg/ml; normal range, 9–52 pg/ml), cortisol [19 μg/dl; normal range (morning), 7–28 μg/dl; normal range (afternoon), 2–18 μg/dl], urinary hydroxymandelic acid (HMA; 253 mg/l; normal range, 21.7±3.2 kg/m^2^) and vanillylmandelic acid (VMA; 3 mg/24 h; normal range, 2–7 mg/24 h) levels were within normal ranges; therefore, the mass was considered to be hormonally inactive. Additionally, biochemical tests were conducted and the results were as follows: Glucose, 90 mg/dl; creatinine, 1.1 mg/dl; urea, 42 mg/dl; white blood cells, 8.2×10^3^/μl; hemoglobin, 13.9 g/dl; and platelet count, 185,000. Mineralocorticoid and glucocorticoid replacement therapy were administered to reduce the risk of developing iatrogenic Addison’s disease. The patient subsequently underwent US-guided biopsy on the renal mass, as well as right radical nephrectomy, right partial adrenalectomy (with frozen section examination) and left adrenalectomy. Postoperatively, cortisol levels were within the normal range and the patient did not exhibit adrenal insufficiency.

### Immunohistopathological findings

**Figure 4:**
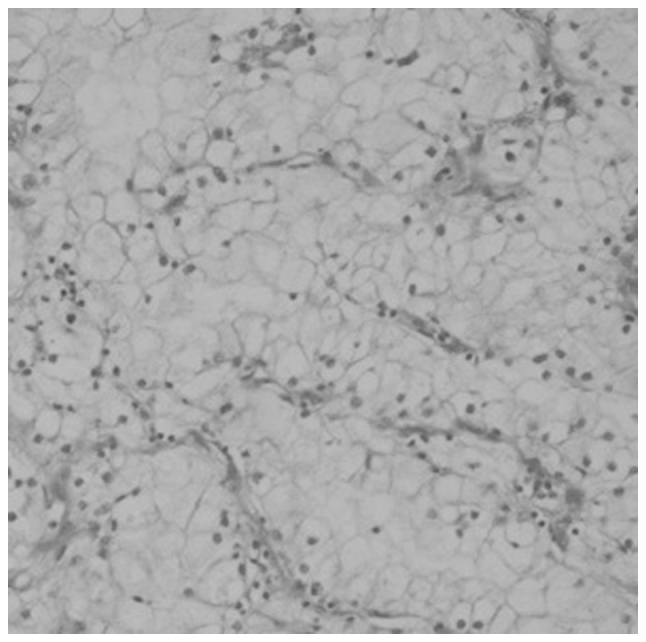
Hematoxylin and eosin staining demonstrating tumor cells with an alveolar structure, clear cytoplasms and small nucleoli (magnification, ×200).

**Figure 5:**
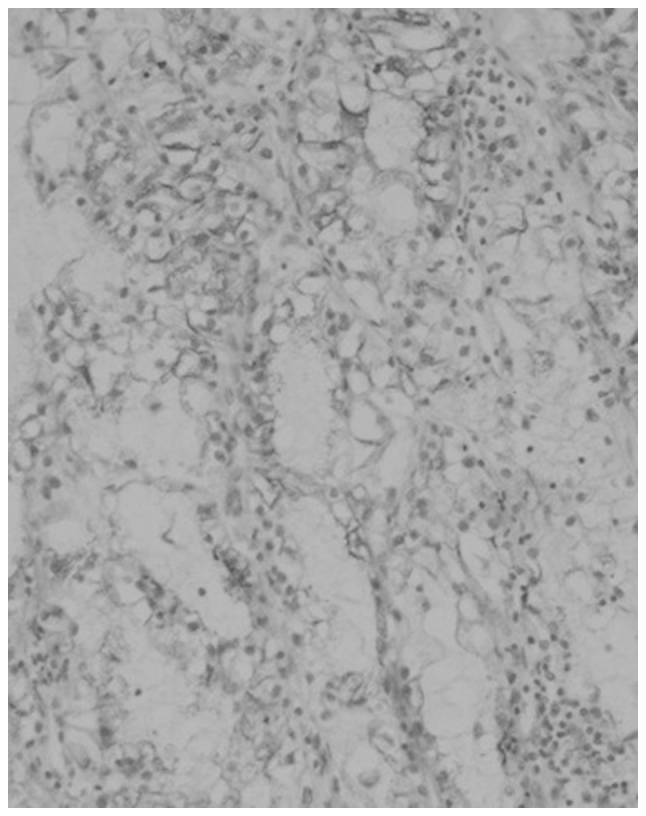
Immunohistochemistry of the metastatic mass demonstrating positive staining for renal cell carcinoma (magnification, ×200).

**Figure 6:**
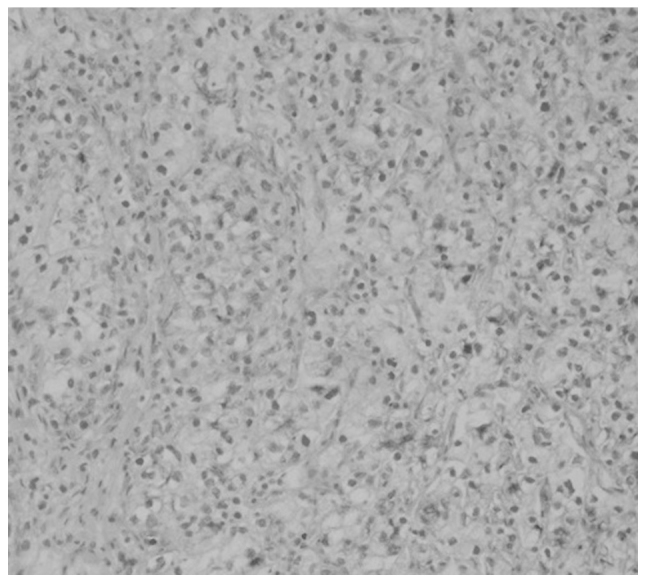
Immunohistochemistry of the metastatic mass demonstrating positive staining for cluster of differentiation 10 (magnification, ×200).

The specimens obtained from the right kidney using the US-guided biopsy technique demonstrated characteristics typical of clear cell RCC, and high magnification examination of the right radical nephrectomy, right partial adrenalectomy and left adrenalectomy specimens revealed tumor cells with an alveolar structure, clear cytoplasm and small nucleolus (hematoxylin and eosin staining; magnification, ×200; Fig. 4). Furthermore, immunohistochemical examination revealed positive staining for RCC and cluster of differentiation 10 (Figs. 5 and 6). Thus, a diagnosis of bilateral adrenal metastasis from clear cell RCC was established.

## Discussion

A routine ipsilateral adrenalectomy is not recommended in cases of radical nephrectomy (6), as there is no difference in the overall survival rate between undergoing surgery and not undergoing surgery. Only 3/2,065 patients who underwent concurrent ipsilateral adrenalectomy exhibited direct invasion of the adrenal gland by RCC or RCC metastasis. Even in the presence of suspicious adrenal lesions detected by imaging techniques, only 13% of these lesions are cancerous (7). Furthermore, in a retrospective study by Kobayashi *et al* (8), no statistically significant difference was identified in terms of cancer-specific survival (CSS) between patients that underwent ipsilateral simultaneous adrenalectomy and patients that underwent adrenal-sparing radical nephrectomy due to RCC. Ipsilateral adrenalectomy does not appear to provide any survival benefits, however, it is currently recommended when upper pole tumors are present, metastases are detected by CT and/or magnetic resonance imaging (MRI), or a renal mass measuring >8 cm is identified (9). Various risk factors have been described for the development of adrenal metastasis, including large renal masses, upper pole tumors, tumors arising from the left kidney and multifocal tumors; however, it must be considered that small lower pole tumors may subsequently develop ipsilateral and contralateral adrenal metastases. These adrenal metastases may occur by tumor spread via the vessel in Gerota’s fascia, the lymphatic vessels, arterial embolism or retrograde venous embolism (10,11). In addition, the risk of developing metastasis is lower in the right adrenal gland compared with the left adrenal gland (12). However, the present case possessed none of the abovementioned risk factors and the burden of metastasis was higher in the right adrenal gland compared with left side. In a systematic review of 11,736 patients conducted by Su *et al* (13), the role of ipsilateral adrenalectomy was evaluated in radical nephrectomy and a novel use for adrenalectomy was proposed. The rate of ipsilateral involvement in RCC was 4.5%; however, upper pole tumors were not associated with a higher incidence of ipsilateral adrenal metastases, and adrenal involvement from RCC was rare, even in advanced tumors. Furthermore, synchronous adrenalectomy did not appear to offer any survival benefit, even for high-risk patients. Therefore, Su *et al* (13) proposed that adrenalectomy should be performed, in addition to radical nephrectomy, when an ipsilateral adrenal mass is detected in the preoperative period.

Although adrenal-sparing radical nephrectomy is a standard surgical procedure, it should not be the preferred method in the presence of suspicious adrenal lesions detected by imaging methods; in this case, adrenalectomy is recommended. A number of cases of RCC with ipsilateral metastasis are considered to be associated with the underdiagnosis of metastasis in the adrenal tissue. In such cases, intraoperative frozen section examination may be of benefit in the decision to perform adrenalectomy (14). Adrenal metastases are common, however, benign adrenal adenomas account for 70% of adrenal masses detected in cancer patients (15). Radiological studies may facilitate preoperative diagnosis; however, they cannot definitively determine whether an adrenal tumor in an RCC patient is a primary adrenal neoplasm, an adrenal cortical adenoma or a metastatic lesion (3). Therefore, a hormonal examination is recommended for RCC patients with adrenal lesions detected that have been detected using CT, MRI or positron-emission tomography/CT (16). This hormonal examination is useful in the differentiation of primary carcinomas of the adrenal gland from other hormonally active tumors. In the present case, the hormonal examination demonstrated that ACTH, cortisol, HMA and VMA levels were within the normal ranges.

The surgical treatment options of for adrenal metastases are similar to those for localized disease (10). Efforts to improve survival are more effective in patients with low metastatic tumor load, good performance status and in those that developed adrenal metastasis late following nephrectomy. Previous studies have demonstrated the long-term survival and palliative benefits of metastasectomy in selected patients (9,10). However, in the current patient, bilateral adrenal metastases were detected synchronously in the early period following nephrectomy. Furthermore, the surgical removal of metastatic foci of RCC in the lungs and bones provides survival benefit (10), and various studies have demonstrated a survival benefit even in the presence of multiple metastatic foci. For example, Alt *et al* (10) identified that metastasectomy provided a survival benefit in patients with RCC and multiple metastatic lesions (9). Similarly, according to data from the Mayo Clinic, complete resection of the metastatic foci provided 4.8 years CSS, whereas incomplete resection was associated with a significant reduction in survival to 1.3 years (11). In metastatic RCC, leaving metastatic foci untreated resulted in a significantly worse prognosis. Instead, surgical removal is the only known effective treatment in patients with solitary adrenal metastasis, resulting in ≥5 years survival in 29–35% of patients (17). In addition, Plawner (18) demonstrated that the five-year survival rate of patients who underwent surgery for metachronous solitary RCC metastases to the contralateral adrenal gland was lower than that for patients with synchronous adrenal metastases (20 and 40%, respectively). The prognosis for RCC patients with adrenal metastases was dependent on the time between the detection of the adrenal metastasis and nephrectomy, and was improved for patients who developed the metastasis late rather than early following nephrectomy. Metastasectomy (adrenalectomy) is particularly recommended to achieve a longer survival period (17). In a previous study of four patients, Chkhotua *et al* (19) identified that the mean cancer specific survival time to the development of adrenal metastasis following surgery was 83.3 months; three patients underwent adrenalectomy and one patient underwent bilateral adrenalectomy, and the authors reported a survival period of 19–63 months. The shortest survival period was achieved in the patient exhibiting bilateral metachronous adrenal metastasis. In the current study, the presence of a bilateral adrenal metastasis was considered to be a poor prognostic factor and the resulting adrenal insufficiency was considered to be an additional reason for the development of comorbidity. Therefore, adrenal-sparing surgical procedures are of particular importance in cases of bilateral adrenal metastasis.

In a previous study, the median survival period for patients with adrenal involvement but no systemic involvement was 11.7 years, however, survival decreased to 16 months in the presence of systemic involvement and to six months following bilateral adrenalectomy (13). The presence of metastatic lesions in both of the adrenal glands (bilateral adrenal metastasis) is regarded as widespread disease (12). Although bilateral adrenalectomy with radical nephrectomy is recommended to decrease tumor load in RCC patients with metastasis to the adrenal glands, patients who underwent this surgical procedure exhibited a poor prognosis. Sparing the right adrenal gland is more straightforward than sparing the left adrenal gland, due to its anatomical position and the low risk of metastasis (12). In the present patient, partial surgery was performed in an attempt to spare the right adrenal gland due to its low tumor load. Parallel to the literature, the right adrenal gland was affected to a lesser extent, despite the primary RCC being located in the right adrenal gland. However, controversial reports exist in the literature, for example, Moudouni *et al* (20) identified that adrenal involvement was a weak prognostic factor in predicting the prognosis of operated RCC. Furthermore, metachronous metastasis of RCC to periadrenal tissue has been described in the literature; however, additional studies are required to develop an adrenal-sparing approach for the treatment of periadrenal tissue metastases (21).

Antonelli *et al* (22) compared lung metastases (LM) from RCC with atypical metastases (AM) from RCC (including, bone, liver and adrenal) in terms of CSS. The study evaluated 1,800 RCC patients, comparing 37 patients who underwent lung metastasectomy with 57 patients who underwent atypical metastasectomy. CSS from metastasectomy was affected by the synchronicity in the diagnosis of metastasis and primary tumor, as well as by the simultaneous presence of other metastases; however, the type of metastasis (AM vs. LM) did not affect the CSS. Notably, metastasectomy in AM was as effective as in LM. The present study emphasizes the importance of RCC metastasectomy regardless of the metastatic location; even in the presence of bilateral adrenal metastasis, adrenal-sparing surgery with the guidance of frozen section examination may provide significant benefits in CSS.

The detection of adrenal metastases, the choice of surgical technique, the perioperative decision process and the challenges in postoperative follow-up produce a clinical picture that is difficult to manage. In the previous study by Antonelli *et al* (22), long-term use of mineralocorticoid and glucocorticoid replacement therapy must have been considered prior to surgery to prevent the possible development of iatrogenic Addison’s disease. Partial adrenalectomy is an ideal treatment strategy, as sparing a section of the adrenal tissue may prevent the occurrence of other comorbid conditions. When intraoperative examination of the frozen sections of the mass and adrenal tissue determine that partial adrenalectomy is not possible, adrenalectomy must be performed. Metastatic lesions in RCC often occur between the cortex and the medulla, however, they can occur at any site within the organ (15). Sparing 10% of the adrenal gland is sufficient for the maintenance of adrenal gland function; therefore, bilateral metastatic spread to both of the adrenal glands rarely causes adrenal insufficiency (23,24). For bilateral metastatic patients, an alternative treatment strategy to bilateral adrenalectomy is partial adrenalectomy, which can be performed in suitable patients to avoid the side effects of steroid replacement therapy, such as gastritis, hypertension and hypokalemia (4,25). In clinical practice, metastasis to the adrenal glands accounts for <1% of patients with primary Addison’s disease (23), however, iatrogenic Addison’s disease occurs relatively more frequently with adrenalectomy. Alkan *et al* (21) recommended robotic surgical approaches in the treatment of adrenal metastases due to lower rate of complications, mortality and morbidity rates, as well as a shorter length of hospital stay and increased patient comfort in the postoperative period (21).

In conclusion, bilateral adrenal metastasis from operated RCC poses a complicated oncological problem, as the optimal diagnostic and treatment strategies of this condition are yet to be thoroughly defined. However, it is of particular importance to detect the hormonal activity of the metastatic adrenal masses and, due to the survival benefits, metastasectomy is important in the treatment of bilateral adrenal metastasis of RCC. If adrenalectomy is required, minimally invasive surgical procedures, such as laparoscopic/robotic procedures, should be preferred and partial adrenalectomy should remain the preferred type of surgery. In cases where adrenalectomy is inevitable, the decision must be based on intra-operative biopsies and frozen section examination. This is due to the necessity of life-long mineralocorticoid and glucocorticoid replacement therapy in patients with iatrogenic Addison’s disease, as well as the lack of adrenal tissue possibly resulting in endocrinological disease, additional to the previous oncological disease.

## Abbreviations

RCC: renal cell carcinoma
US: ultrasound
CT: computed tomography
MRI: magnetic resonance imaging
ACTH: adrenocorticotropic hormone
HMA: hydroxymandelic acid
VMA: vanillyl mandelic acid
CSS: cancer-specific survival
